# Cycles and the Qualitative Evolution of Chemical Systems

**DOI:** 10.1371/journal.pone.0045772

**Published:** 2012-10-11

**Authors:** Peter Kreyssig, Gabi Escuela, Bryan Reynaert, Tomas Veloz, Bashar Ibrahim, Peter Dittrich

**Affiliations:** 1 Bio Systems Analysis Group, Institute of Computer Science, Jena Centre for Bioinformatics and Friedrich Schiller University, Jena, Germany; 2 Faculty of Biological Sciences, University of Chile, Santiago, Chile; 3 Department of Psychology and Computer Science, University of British Columbia, Kelowna, Canada; 4 German Cancer Research Center, DKFZ-ZMBH Alliance, Heidelberg, Germany; Institut de Génétique et Développement de Rennes, France

## Abstract

Cycles are abundant in most kinds of networks, especially in biological ones. Here, we investigate their role in the evolution of a chemical reaction system from one self-sustaining composition of molecular species to another and their influence on the stability of these compositions. While it is accepted that, from a topological standpoint, they enhance network robustness, the consequence of cycles to the dynamics are not well understood. In a former study, we developed a necessary criterion for the existence of a fixed point, which is purely based on topological properties of the network. The structures of interest we identified were a generalization of closed autocatalytic sets, called chemical organizations. Here, we show that the existence of these chemical organizations and therefore steady states is linked to the existence of cycles. Importantly, we provide a criterion for a qualitative transition, namely a transition from one self-sustaining set of molecular species to another via the introduction of a cycle. Because results purely based on topology do not yield sufficient conditions for dynamic properties, e.g. stability, other tools must be employed, such as analysis via ordinary differential equations. Hence, we study a special case, namely a particular type of reflexive autocatalytic network. Applications for this can be found in nature, and we give a detailed account of the mitotic spindle assembly and spindle position checkpoints. From our analysis, we conclude that the positive feedback provided by these networks' cycles ensures the existence of a stable positive fixed point. Additionally, we use a genome-scale network model of the *Escherichia coli* sugar metabolism to illustrate our findings. In summary, our results suggest that the qualitative evolution of chemical systems requires the addition and elimination of cycles.

## Introduction

Many mechanisms are characterized by the presence of cycles, especially those that play central roles in biological systems. Specific functions such as regulation, memory and differentiation have been associated to cycles [1]–[4]. Furthermore, systems containing cycles exhibit robustness to environmental changes, which is a key feature for the evolution of biochemical networks [5], [6]. Cycles vary in appearance from simple feedback loops to coupled ones [7] or large cycles, these include some signaling cascades [8], and the Krebs and Calvin cycles [9], [10]. Frequently, simple cycles are considered as network motifs [11] and therefore, they are analyzed as isolated modules neglecting the role of the molecular environment. In contrast, it has been proposed that the topological structure of a subgraph alone cannot determine its effects over the whole network [7], [12]. Moreover, some authors have found interesting results on feedback loops and have concluded these as prerequisites for multistability in gene regulation and mixed networks [1], [2], as well as in metabolic networks [4], [13]. However, the existence of cycles as well as their necessity and contribution to stability in networks remains largely elusive. Additionally the question of how to analyze large systems, in which classical approaches like differential equations fail, is open.

Chemical organization theory (COT) offers a lucid formalism with novel methods to analyze complex systems and their dynamics at specific states. It can be particularly useful for analyzing large scale models and can be applied in a broad range of disciplines such as political, social, biological and chemical systems. COT has been developed for the last 20 years [14]–[16] and right from the beginning it has focused on two central notions, namely the existence of closure and self-maintenance. These notions contribute to the further understanding and characterization of relevant systemic properties, since COT developed a relation between both topological and dynamical aspects of reaction networks [17].

On the other hand, reflexive autocatalytic food-generated (RAF) sets theory [18] is another formal framework, which contains some indications on the necessity of the existence of a cycle [13] for the stability of living systems. A RAF set is a set of molecules which can be produced from an initial set of food molecules in a reflexive autocatalytic way, i.e. every reaction is catalysed by at least one molecular species. RAF sets and COT share similarities as they are based on set theory and even coincide in some definitions, e.g. closure. The main difference between these formalisms resides on how dynamics are approached, since RAF sets place catalysis as the only element concerned with kinetics and occurrence of reactions. The requirements that every reaction should be catalysed and all catalysts should come from within the reaction network is very strong. However it is sensible since many networks in biology fulfill this condition. In contrast, COT focuses on the connections between the stationary states and the topological and stoichiometric properties of the reaction network [13], [17] while making less restrictive hypotheses, especially none on the structure of the reactions directly. This leads to a greater generality.

We give a definition of cycles in reaction networks based on a directed graph extracted from the reaction network (Definition 1). Using this definition, we prove the existence of a cycle to be a necessary condition of non-trivial stationary states in reaction networks. This is our main result, formulated as Lemma 1. It also yields a potential way to create or alter a non-zero stationary state of a system by adding a cycle to a closed, but not necessarily self-maintaining, set of molecules. Thereby the system is changed qualitatively since in the new fixed point a different composition of molecular species is present.

When employing this COT-based analysis for systems that contain cycles, we found that there are some interesting properties that can be easily deduced, including the relation between the hierarchical structure of the set of chemical organizations and the existence of cycles. Moreover, in this work we rediscover the abundance of cycles in biological systems. Considering the BioModels Database [19], we see that more than 60% of the models have more than one organization [20], and thus we can deduce from Lemma 0 that they contain cycles. This insight encourages us to believe that COT will be able to contribute to the further understanding of cycles in reaction networks. Hence we focus on the combination of COT with results about the existence of cycles (i.e. an overlay of COT and cycles).

We apply our main result to four examples of different nature. Firstly, we analyze the cycle found in a particular autocatalytic network, which is a generalization of the system discussed in [13], and investigate its stability properties further using COT, the Jacobian and numerical simulations. Secondly and thirdly, in order to provide some realistic examples for such autocatalytic networks, we discuss two real biological models of mitotic control mechanisms in detail. One of these has already been published [21] and one was newly constructed for this study. These examples support our ideas and illustrate how they can be used to study biological systems. Fourthly, we further investigate the consequences of Lemma 1 for a large network, namely the sugar metabolism model proposed in [22].

## Methods

We shortly summarize the needed definitions and results from COT in informal terms, still providing a mathematically precise version in the Appendix. A tool for the computation of chemical organizations is freely available on our website http://www.biosys.uni-jena.de/Services.html.

The pair 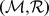 is called *reaction network* and we call 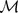 the set of *molecules* (or *species*) and 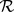 the set of *reactions*. Each reaction 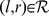 consists of a left hand side 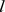 and right hand side 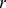 and will be denoted also by 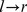 in concordance with the chemical vocabulary. A reaction 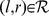 *occurs* when the molecules in 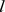 are *consumed* to *produce* the molecules in 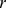 (what this actually means has to be made precise for every dynamics separately, e.g. the application of a reaction in stochastic dynamics is different from continuous dynamics).

**Figure 1:**
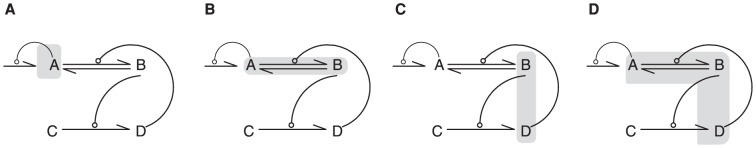
Example for the definitions of reaction networks and cycles. There are four cycles in this example network, given by 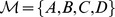 and 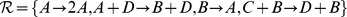, shown in shaded areas. Panels A to D show cycles 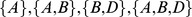 respectively. The arrow 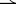 refers to a reaction while the arrow 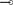 indicates the catalytic effect on a reaction.

Figure 1 depicts the network 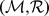 consisting of the set of species 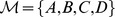 and the set of reactions 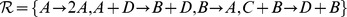.

Furthermore we define 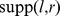, the *support*, and 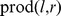, the *product*, of 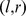 to be the set of species occurring on the left hand side and on the right hand side, respectively. We emphasize that the support and product are sets, meaning that the *multiplicity* of each molecular type in the reaction does not matter. The sets 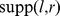 and 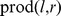 only collect the species that are present (i.e. have a multiplicity greater or equal than one) on the left and right hand side respectively. In our example this means that the product of the first reaction 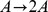 is the set 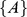 and not the multiset 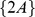. Unfortunately the notion of a support with the same notation is also used in RAF sets theory [13], [18] for all the molecules on both left hand side and right hand side. Within this article the usage of 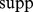 is consistent with COT.

Let 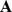 be a subset of 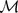. We define 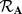 as the set of reactions applicable to 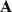, or in other words the reactions using only species from 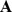. Using this terminology we define a subset 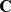 to be closed if by applying reactions from 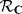 we do not get molecules outside 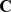. In other words, in a reactor containing molecules from 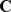, we will never find molecules not already in 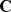. The set 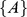 in the example is therefore closed. If we only have molecules of type 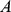 there is no reaction producing new species (for the reaction 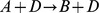 to be applicable, for example, we would need molecules of type 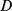 as well). Also we call 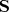 *semi-self-maintaining* if a reaction application consumes a species, there is a reaction producing this species. Combining these two definitions we arrive at the notion of a *semi-organization* which is a closed and semi-self-maintaining set.

Semi-organizations entail a topological (non-stoichiometric) notion of stability in reaction networks. On the one hand, the closure property ensures that a semi-organization will not produce novel species. On the other hand the semi-self-maintenance ensures that the species that are consumed within the network are also produced. However, although semi-organization is a necessary condition for the occurrence of a fixed point, semi-organizations does not necessarily ensure permanence, i.e. consider the reaction network 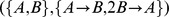 with the semi-organization 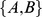.

In order to capture more of the notion of fixed states in a reaction network, we need to consider the stoichiometry of the reactions, and verify if the network can maintain itself, i.e. produce all the species at a higher or equal rate to what they are consumed. The *stoichiometric matrix* represents the numerical relation between production and consumption of species in a reaction network, and it is denoted as 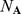, where 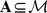. 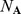 is calculated taking the difference between the right and left hand sides of each reaction in 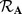.

We say 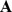 is *self-maintaining* if and only if all the reactions from 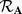 can occur at a certain strictly positive rate without decreasing the concentration of any species of 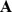. A subset of 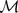 is a *chemical organization* if it is closed and self-maintaining [14], [16].

The term organization refers to a more stringent condition than semi-organization. Indeed, every organization is a semi-organization [16]. Moreover, organizations bridge the reaction network with its dynamical behavior. Given a reaction network and a kinetic law (e.g. mass action kinetics), it has been proven that the fixed points of the obtained ordinary differential equation (ODE) system, relate to the set of organizations as follows. For every fixed point the set of molecules with concentration higher than zero in that fixed point is an organization of the reaction network [16], [17].

In general, an organization may contain a species that does not take place in any reaction within this organization. Hence, it is reasonable to introduce the notion of a *reactive set* of species. A set 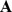 of species is reactive if and only if for all 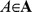 there exists a reaction 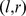 in 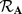 such that 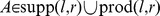. Note that the dynamical properties of a set of species depends only on its largest reactive set, as the other species do not react and remain with constant concentration. Furthermore, the number of organizations in most reaction networks is considerably larger than the number of reactive organizations, and hence focusing on the set of reactive organizations instead of the whole set of organizations simplifies the understanding of the dynamical behavior of a reaction network [23]. From now on, we will consider only reactive sets of species.

## Results

Our definition of cycle can be explained using the usual definition for cycles in directed graphs. Given a reaction network we can deduce a directed graph in the following way. The nodes of that graph are the species of the reaction network, and there is an edge from species 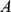 to species 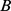 if there is a reaction with 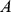 on its left hand side and 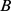 on its right hand side. We say that 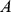 is directly-causally connected to 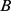. The cycles found in this directed graph are the ones we identify as cycles of the reaction network in our terminology (see Figure 1). This definition captures a variety of specific forms of cycles, yet yielding a structural result about reaction networks.

### Definition 1

*Two species* 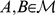 *are* directly-causally connected, *if there exist a reaction* 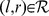 *with* 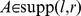 *and* 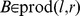 *, i.e.* 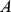 *is on the left hand side of a rule and* 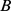 *on its right hand side. We write* 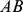 *. We say that the network contains a cycle if there is a subset of species* 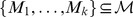 *such that* 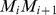 *for* 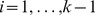 *and* 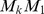 *. We write* 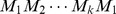 *and say that* 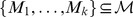 *is a cycle.*

We can now formulate our main theoretical result:

### Lemma 1

*Given a reaction network and a reactive semi-self-maintaining set* 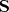 *that strictly contains a closed set* 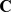 *, i.e.* 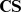 *, then* 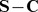 *contains a cycle*.

#### Proof

Let 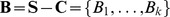 for some 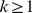 as 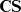. We will prove first that at least one reaction in 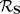 consumes species from 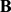. Suppose that no species in 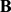 is consumed by any reaction in 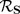. As 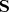 is reactive, then each species 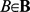 must be produced by some reaction 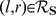. However, as no species in 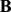 is consumed, we have that either 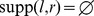 or 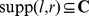. This contradicts the hypothesis that 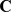 is closed, since elements from 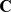 would produce an element which is not in 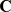. Hence, at least one species, say 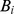, is consumed within 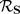.

As 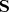 is semi-self-maintaining, 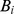 is produced by a reaction 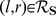. Moreover, 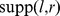 must contain at least one species 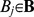 since 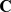 is closed. There are two possible cases.

- First case: 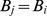. By definition 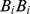 and we have a (trivial) cycle.
- Second case: 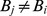. By definition we have 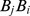.

**Figure 2:**
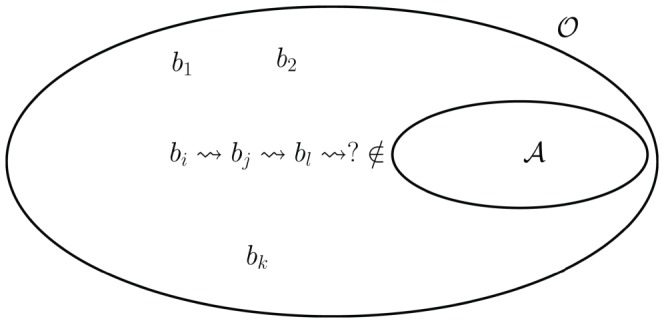
Situation in the recursion argument of the proof of Lemma 1.

Since the same argument applies for 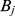 as well, by a recursion argument we prove that there exists a cycle (see Figure 2 for a schematic representation). 

Lemma 1 connects the existence of reactive semi-self-maintaining sets with the existence of cycles. It states that every reactive semi-self-maintaining set that strictly contains a closed set must contain a cycle made from molecules not contained in the closed set. Why do we require that there is a closed set? Let us consider the example 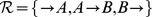. The set 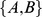 is self-maintaining, but does not contain a cycle. Note that it does not strictly contain any closed subset, because the closure of the empty set is 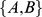. Since the closure of the empty set is contained in all closed sets we can say that a reactive semi-self-maintaining set whose molecules cannot be made from the inflow must contain a cycle.

Note that every organization is semi-self-maintaining and closed. Thus, equating in Lemma 0 the closed set 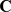 with an organization 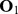 and the reactive semi-self-maintaining set 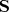 with a reactive organization 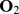 we arrive at the following corollary.

### Corollary

*Given a reaction network and two different reactive organizations* 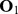 *above* 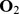 *, i.e.* 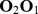 *, then* 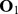 *contains at least one more cycle than* 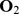.

From Corollary 1 follows that any reactive organization, except the ones produced from the inflow of the network, contains at least one cycle. Or in other words only the smallest might not contain a cycle, like in the previous example. The shifting or moving from one reactive organization and therefore from one potentially stable constellation of molecules to another was already described in [24]. According to Lemma 0 this movement must involve the addition or removal of at least one cycle. In particular this can be achieved in two ways. Combining the species of two organizations with disjoint cycles is the first possibility. The second is adding one or more species to an organization to create new cycle. As an example we refer to Section “A Particular Autocatalytic Network” The reverse operations lead to the “destruction” of organizations. Thus, any qualitative transition from a stable state to another with a different composition of reacting molecular species must encompass the removal or addition of a cycle.

Note that Corollary 1 is also true for *overproducible* organizations, i.e. organizations where each species can be produced at a strictly positive rate, because overproducible organizations are reactive sets.

Moreover, from Corollary 1 we can also infer that every organization is uniquely identified by its set of cycles, and if the set of cycles of an organization 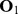 contains the set of cycles of another organization 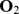, then 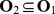. This implies that the hierarchy of the set of organizations [16] can be mapped to a hierarchy of the set of cycles.

Considering RAF set theory, not every RAO set is necessarily a reactive set. The other hypothesis is satisfied by all RAOs though, since for every catalyst 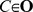 the subset 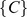 is closed. Therefore Lemma 0 and the cycle theorem proven in [13] are complementary results.

### Theoretical and Biological Applications

#### A Particular Autocatalytic Network

**Figure 3:**
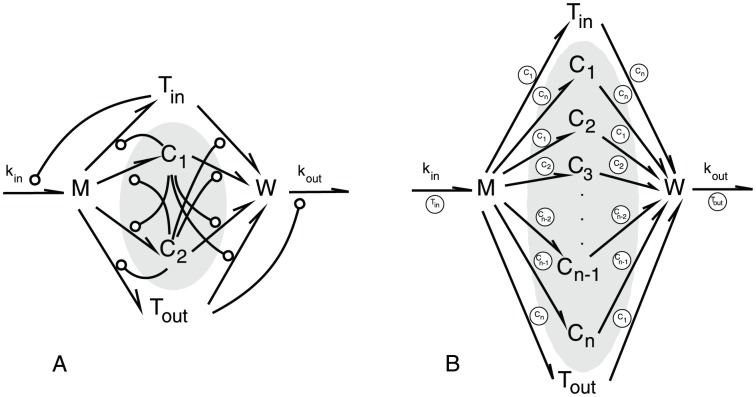
Particular autocatalytic network. (A) Original network presented in [13] and (B) a generalization, used for our analysis. 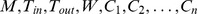 represent species. Arrows indicate reactions between the species, arrows ending with a circle denote catalysis (to reduce its complexity, in Panel (B) the catalysts but not the arrows are shown). The parameters 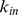 and 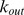 represent reaction rates. Observe that 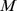 and 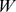 are the external supplies and waste species, 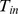 and 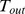 represent transport molecules, whereas 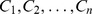 conform a cycle, i.e. 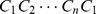.

In this section we apply Lemma 1 and show how our algebraic analysis can be complemented by a dynamical analysis. We introduce a prototypical model (Figure 3) that captures properties of reaction networks found in origin of life and protocell research [25]. This model is a generalization of the RAF model by Contreras et al [13]. It incorporates a basic metabolism built from enzymatic reactions and includes some environment variables for the transport, production and decomposition of the species. Hence the environment, even if in a relatively simple way, is considered. Furthermore we consider five variants of our generalization (case 1). Since in biological applications there are often also reversible reaction given, i.e. due to thermodynamics, the network in case 3 incorporates reversible reactions for all the reaction given in the network. However not all reactions in biological models need to be reversible [19]. Hence we also consider a model in which one of the reactions is not reversible (case 5). In order to examine the effect of the positive feedback given by the cycle we took, without loss of generality, one reaction out of the set of possible reactions from the networks in cases 1, 3, 5. This constitutes the networks 2, 4, 6 respectively.

**Figure 4:**
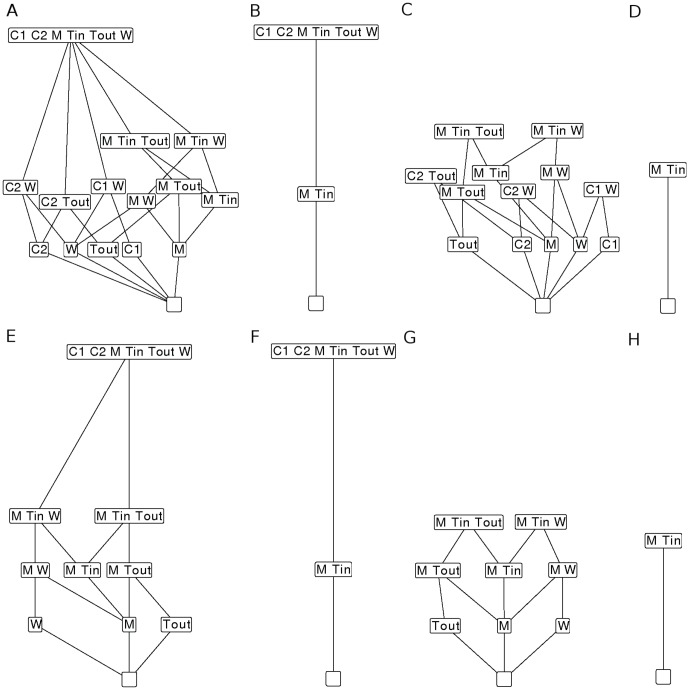
Hasse diagrams of organizations for the 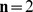
 **case of the particular autocatalytic network with and without reversible reactions.** A and C show the Hasse diagrams of the organizations with feedback (closed cycle) and without feedback (open cycle) respectively for the particular autocatalytic network without reversible reactions. When adding reversible reactions, we have panels E and G which show the Hasse diagrams of the organizations with feedback (closed cycle) and without feedback (open cycle) respectively. In B, D, F and H we show only reactive organizations of the panels A, C, E and G respectively (see Section “Methods”). For example 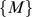 is an organization in both cases, feedback and no feedback, but 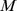 alone does not react to anything. Hence it is not a reactive organization.

Figure 4 illustrates how our main result, Lemma 1, can be applied to analyze the particular autocatalytic network model Figure 3. This network has three reactive organizations (Figure 4B). The smallest organization does not contain any molecule and thus also no cycles. According to the main result Lemma 1 all other reactive organizations must contain cycles. First, the organization 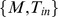 emerges by adding the trivial cycle 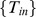 (Figure 4B). Second, the largest organization contains a cycle with the catalysts 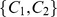 (Figure 4B). The catalytic effect is necessary for the organizations to exist. If we remove the cycle's catalytic effect, the largest organization vanishes, as can be seen in the reactive organizations of the network with the cycle ( case 1, Figure 4B) compared the ones of the network without the cycle ( case 2, Figure 4D). Due to the special structure of the model considered here we are able to provide a further analysis of dynamical aspects using ODEs.

In order to define the particular autocatalytic network under consideration, we use the molecular species
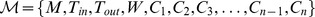
and the set of reactions
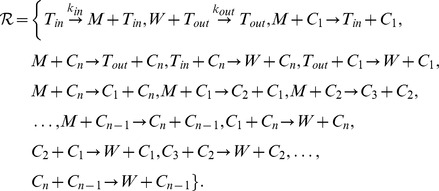
The set of reverse reactions is
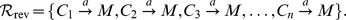
We decided to use reaction rates equal to 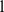 in most of the reactions in order to have a simple and usable model. Also the main influence of reaction rates should be given by the catalysts as suggested in RAF set theory. The parameter 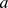 gives control over the strength of the reverse reactions and 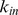 and 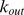 govern the inflow and degradation, respectively.

We will focus on the following reaction networks:

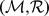
 – generalized cycle

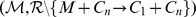
 – generalized open cycle (the feedback is missing)

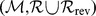
 – generalized cycle with reversible reactions

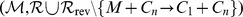
 – generalized open cycle with reversible reactions (the feedback is missing)

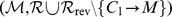
 – generalized cycle with all reactions reversible except of one

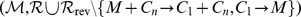
 – generalized open cycle with all reactions reversible (the feedback is missing) except of one

It is possible to compute the fixed points and to analyze their stability analytically in the first case. The analysis with the help of chemical organizations yields that there cannot be a positive fixed point in the cases 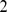, 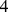, 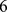. The other cases 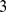, 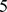 can only be treated numerically (analytical solution was not found using Mathematica [26]) but yield similar results and are more realistic for biological applications.

**Figure 5:**
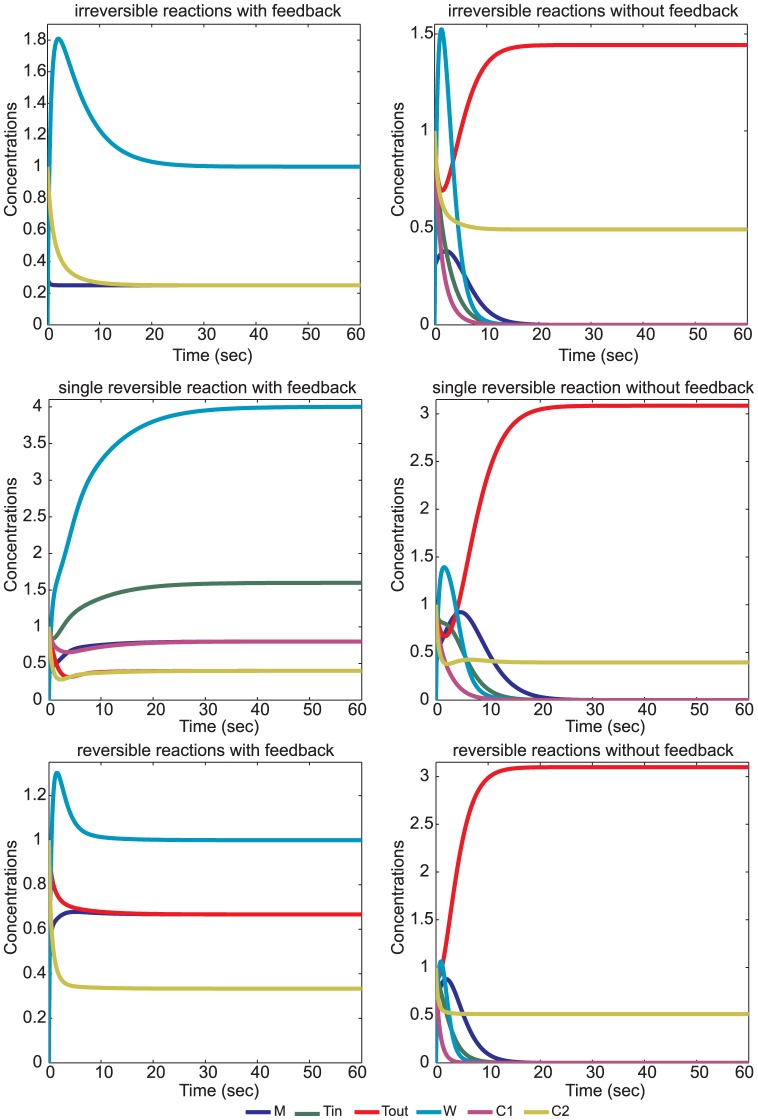
Simulations of all particular autocatalytic network variants (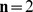) with and without feedback. We chose initial conditions as follows: 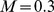, 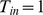, 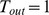, 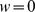, 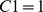, 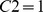. All forward reaction coefficients are set to 1 while reversible rates are set to 0.1. We tested the effect of different relative reversible rates and we found that they have no influence on the systems (see Appendix “Fixed Point and Stability Analysis for the Particular Autocatalytic Network” ) for details.

In summary we see that the three reaction networks 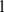, 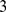, 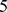 with a cycle have a stable positive fixed point whereas the networks 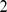, 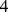, 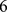 without the feedback via the cycle do not exhibit a positive fixed point at all. So the feedback of the cycle ensures the existence and stability of a fixed point. Numerical simulations of all particular autocatalytic network variants are shown in Figure 5. A detailed account is provided in the Appendix.

#### Mitotic Spindle Assembly Checkpoint Network Model

**Figure 6:**
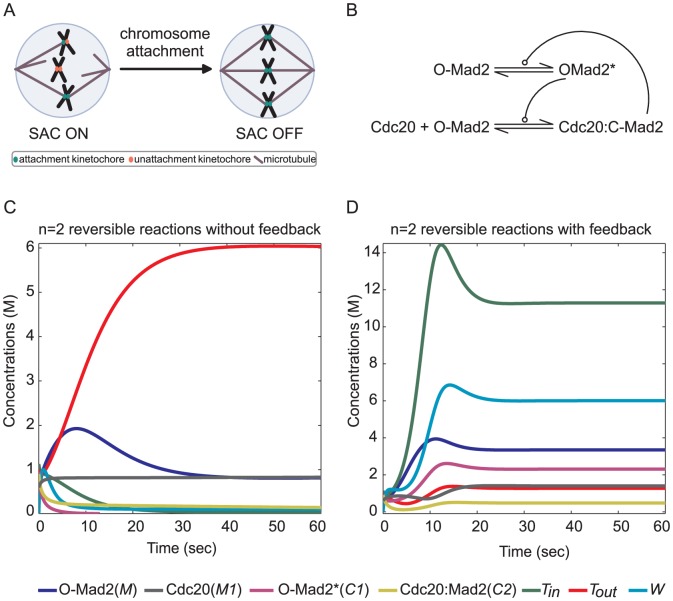
The Template model of the mitotic spindle assembly checkpoint (SAC) mechanism. (A) Pictorial representation of the SAC mechanism. (B) The biochemical reaction of the Template model which is the interface of SAC mechanism. We added the species 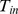, 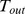 and 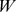 to this core model so that we end up with the particular autocatalytic network Figure 3A, where the species 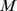, 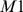, 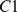 and 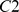 refer to O-Mad2, Cdc20, O-Mad2* and Cdc20:Mad2 respectively. (C) Numerical simulations of ordinary differential equations where all species concentrations are presented. All species reach steady state after about 30 seconds. (D) Same as in (C) after removing the positive feedback from Cdc20:C-Mad2 to activate O-Mad2 (refers to Mad2*).

The mitotic Spindle Assembly Checkpoint (SAC) ensures accurate chromosome segregation by restraining cell-cycle progression from entering anaphase until all chromosomes have made proper bipolar attachments to the mitotic spindle (Figure 6A). It is thought that unattached or misaligned kinetochores catalyze the formation of a “wait-anaphase” signal which then diffuses to counter the activation of the ubiquitin ligase APC by its coactivator Cdc20. Activation of APC by Cdc20 triggers chromosome segregation by ubiquitination of securin and cyclin B. Dysfunction of the SAC leads to aneuploidy [27], [28] and its reliable function is important for tumor suppression [29], [30].

The Cdc20-binding protein Mad2 was suggested as playing crucial and major part in the “wait-anaphase” signal, as it is stabilized in a conformation with increased affinity to Cdc20 specifically at unattached kinetochores. This module was called the “Template” model [31]. According to this model, Mad2 in its open conformation (O-Mad2) is recruited to unattached kinetochores by Mad1-bound Mad2 in its close conformation (C-Mad2) to form the ternary complex Mad1:C-Mad2:O-Mad2*. In this complex O-Mad2* is the “activated” Mad2, i.e. it is stabilized in a conformation which can interact with Cdc20 to form Cdc20:C-Mad2. It has been proposed [32] that there exists an autocatalytic amplification (or a cycle, according to our definition) of Cdc20:C-Mad2 formation by which in addition to the activation via kinetochore-bound Mad1:C-Mad2, O-Mad2 can likewise be activated by Cdc20:C-Mad2 (Figure 6B).

The dynamics of the full Template model both *in-silico* and *in-vitro* have been recently discoursed in detail by several mathematical models [33]–[35]. In this study, we first reduce the Template model to solely two reversible reactions with an autocatalytic amplification which is suggested in [31]. This was observed *in vitro* [35] and has been widely discussed in [36], [37]. Subsequently, we incorporate it into the particular autocatalytic network model presented in Section “A Particular Autocatalytic Network” by adding species and reactions forming production and degradation pathways for all species (compare Figure 6B with Figure 3A). This is justified since we know that all proteins are synthesized during the cell-cycle and at least some are degraded [38]. In contrast, all models in the literature [21], [33]–[35], [39], [40] did not consider the production and degradation of Mad2 and Cdc20. We used arbitrary values of the kinetic parameters and avoid using published or optimized kinetic data from Budding yeast [35] or humans [31], [34] because our approach indicates that the kinetic parameters for the small cycles play no role (see last section of Appendix) and our model is a reduction of the published models where the current reactions have unknow values. We stopped the simulations after one minute which was enough to reach the steady state for the system (Figure 6C and Figure 6D). This time can be thought of as the period required for attaching a single kinetochore to the microtubule (e.g. in humans during mitosis, we have 92 kinetochores). The simulation analysis is in the same concert as our theory where the model has no positive fixed point in the absence of a positive feedback provided by a cycle (Figure 6D) while in the presence of the cycle the system has a stable positive fixed point (Figure 6C). Only the positive fixed points are relevant for our given biological application, since we want all the species to be maintained. Hence our simple model of SAC supports the idea that the autocatalytic amplification (or cycle) is necessary for the topology of the Template model.

#### Spindle Position Checkpoint Network Model

**Figure 7:**
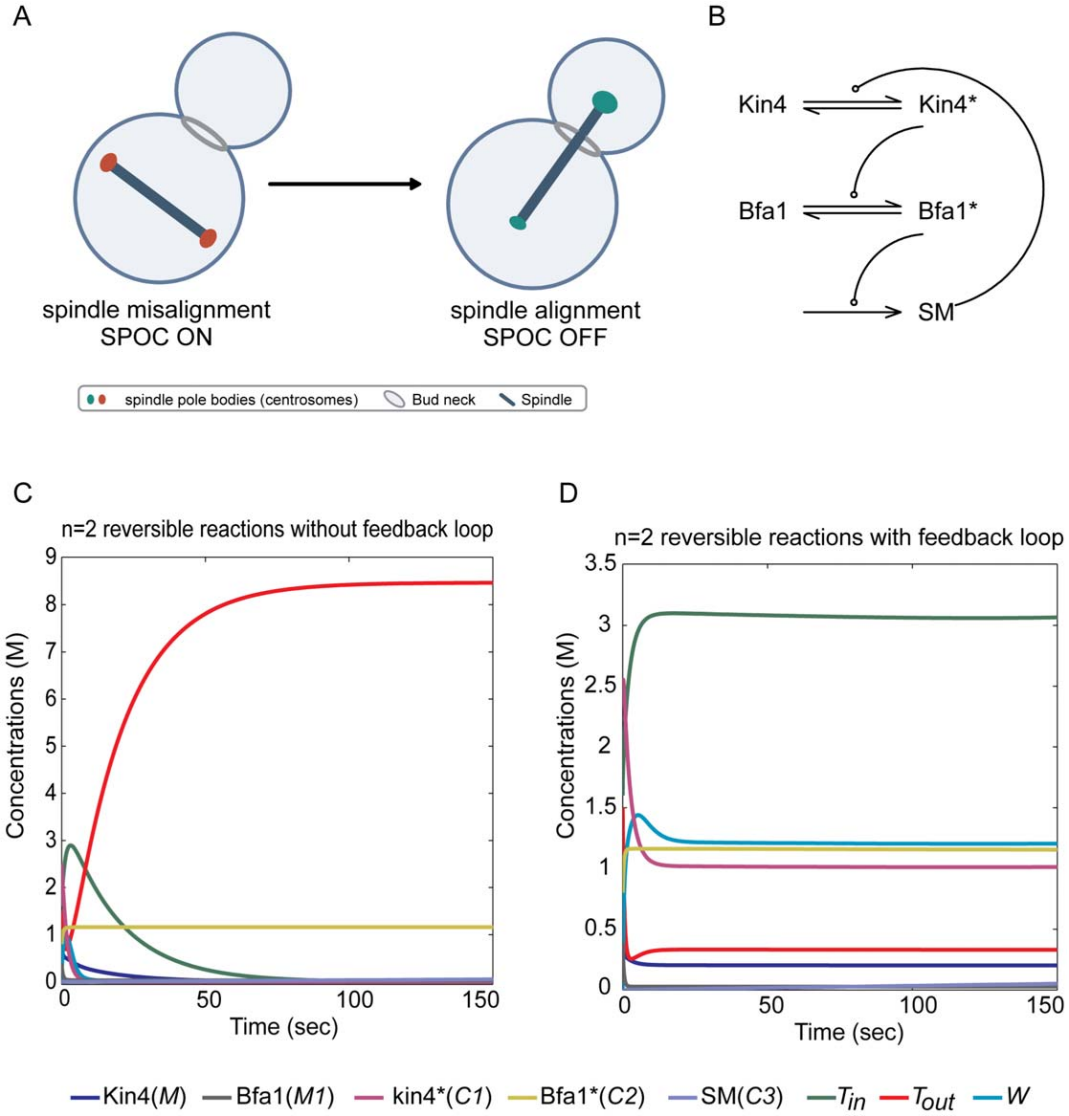
The spindle position checkpoint in budding yeast (SPOC). (A) Pictorial representation of the SPOC mechanism. (B) A rather simplified model for SPOC mechanism. In response to mis-orientations, Bfa1 is activated through Kin4 kinase. Active Bfa1 preventing mitotic exist and enhance SM signaling. The later regulate Kin4 activations. We added the species 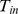, 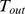 and 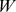 to this mechanism so that we end up with the generalized particular autocatalytic network Figure 3B, where the species 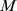, 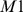, 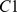, 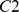 and 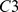 refer to Kin4, Bfa1, Kin4*, Bfa1* and SM respectively. (C) Biochemical reactions in (B) have been translated to mathematical language of ODEs and simulated without positive feedback. Almost all species are famishing after 40 seconds. (D) same as in (C) with positive feedback presented.

Spindle orientation with respect to the polarity axis is crucial during asymmetric cell division [41]–[45]. The budding yeast *S. cerevisiae* is a unicellular organism which undergoes asymmetric cell division and has been widely used to study polarized cell growth and asymmetric cell division [46]. If the spindle fails to align properly, a remarkable surveillance mechanism called the spindle position checkpoint (SPOC) (Figure 7A), delays cells from exiting mitosis until correct spindle orientation is achieved [46]–[49]. SPOC keeps the activity of the Bfa1-Bub2 GAP complex under tight control. Upon spindle misalignment, the kinase Kin4 phosphorylates Bfa1, preventing its inhibitory phosphorylation by another kinase called Cdc5. Kin4 is therefore crucial for maintaining the GAP complex active [46], [50], [51]. Here, we build a simple mathematical model for SPOC active state. Analogously to the SAC model, we incorporated the cycle (Figure 7B) into the structure of the particular autocatalytic network (Figure 3B). Our aim is to see the effect of the positive feedback given by the cycle when we include a production and a degradation pathway for all species. The simulations show that the closed cycle (the signal SM that targets the kinase Kin4) leads to a stable positive fixed point whereas the open cycle does not (compare Figure 7D and Figure 7C).

#### Genome-Scale Network

In an earlier study the organizations of four versions of a genome-scale metabolic reaction network model 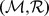 of *Escherichia coli* [22] were already computed [23]. The fourth of the scenarios consists of 762 species and 1939 reactions, where reversible reactions are counted as two single reactions. It exhibits four reactive organizations 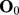, 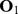, 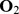 and 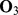 of which the biggest one 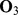 contains 547 species.

For this study we developed a Java tool (available at http://www.biosys.uni-jena.de/Services.html ). to find cycles. Our theory predicts the existence of at least one additional or changed cycle if we look at the difference between two reactive organizations. The results are summarized in Tables 1, 2 and 3. In the following we only describe our analysis for the newly appearing cycles in the largest (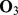) compared to the second largest reactive organization (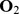) in detail. There is one large cycle 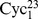 and a small one 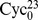 consisting of only two species. In the following we only consider the large cycle since the small one has no apparent influence on the system as a whole. The large cycle consists of 523 different species and there are 913 different reactions constituting it.

**Table 1:** Number of species of the reactive organizations.

	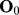	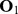	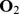	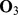
species	31	487	532	547

We number the reactive organizations by size in ascending order from 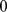 to 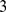.

**Table 2:** Number of species and reactions in the genome-scale network and the cycles.

	*M*	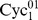	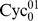	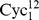	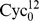	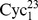	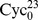
species	762	463	2	599	2	523	2
reactions	1939	837	2	899	2	913	2

*M* denotes the full network, 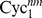 the bigger of the two newly appearing cycles between 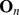 and 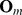, 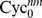 the smaller one.

**Table 3:** Critical and non-critical reactions in the large cycle compared to the ones outside the cycle.

	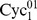	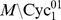	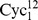	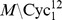	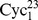	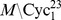
total reactions	837	1102	899	1040	913	1026
critical reactions	353	13	386	11	400	11
non-critical reactions	484	1089	513	1029	513	1015

In order to measure how important the cycle is for the system to be able to maintain a high amount of different species, namely for a potential fixed point to exist, we performed the following experiment. For each reaction in the cycle we check whether the largest organization still exists in the network with the reaction switched off. The same procedure is applied to the reactions not part of the cycle.

In summary, we can say that 400 of the 913 reactions being part of the cycle were identified as crucial for the presence of the largest organization. If one of these reactions is switched off the largest organization cannot be found. In case of the reactions outside the cycle, only 11 of 1026 of reactions are crucial for the existence of the organization. This indicates that the structure of the cycle is important for the existence of the stable composition of species formed by the largest organization.

The runtime of our measurement is neglectable compared to the problem of finding the reactive organizations in such large networks [52]. After a preparatory step to construct a graph from the reaction network, we use Tarjan's algorithm for detecting the strongly connected components of a graph [53] to find our cycles. Both steps have a runtime of 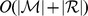. In order to check whether a set of molecules 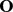 remains an organization when a reaction is switched off, we need to solve a linear programming problem of size 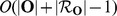. This is done using lpsolve [54].

## Discussion

The occurrence of specific motifs and their functionality in networks have been studied largely in biological, ecological and social systems [11]. Moreover, there are many works that present results, linking reaction networks with cyclic structures and the capacity to generate stationary states [1], [2], [4]. These studies either focus on isolated specific motifs or on the effect of feedback loops on a system's behavior. Here however, we focus on the role of cycles in systems that undergo a change in the composition of molecular species. In Lemma 1 we showed that one possibility to achieve a qualitative transition, namely from a closed (not necessarily self-maintaining) set to an organization, is to add a cycle.

On the one hand, this transition can be interpreted as a way of creating an autopoietic system from a non-autopoietic one. A chemical reaction system in which the present molecules are not able to react such that new species occur (as stated in our definition of closure) may not be sufficient to have an autopoietic property. In origins of life research one is interested in the transition from non-living to living chemical systems. Our results suggest that the addition of a cycle is a necessary condition. Furthermore the evolution from one autopoietic system to another one then includes the additions, changes and/or deletions of cycles.

On the other hand, a change of composition of molecular species in a reaction network also gives rise to new behavior and in particular to new fixed points or a change in the stability of already existing ones. In terms of COT, altering the present molecular species means a movement between organizations. The results on cycles suggest two ways of how a system moves from one organization to another and therefore potentially between stable states. Namely, a cycle can be added or eliminated (see Results section). The results presented here (Lemma 1 and its Corollary 1) provide a framework to study how the inner and outer perturbations lead to qualitative changes in the composition of a chemical system. We emphasize, that this is not only a change of state going from stable state to another one, but a change in the molecular species present at the stable state. As shown in the example on the *E. coli* sugar metabolism model this analysis can even be employed in cases where ODEs fail to predict properties. In particular, it indicates how to identify reactions and species responsible for the stability of the dynamical behavior of the network. Furthermore, organizations can be decomposed in groups of self-maintaining clusters, that are connected through catalysts and overproducible molecules [55] and hence give more insight into the systems' dynamical structure.

An examination of qualitative properties like fixed points and stability of cycles has not been a subject in the mentioned studies. Also COT can only disprove the existence of a fixed point but not prove it. Therefore we need to use classical methods (like the Jacobian). This task can be performed for concrete classes of networks only. We took advantage of an already existing prototypical autocatalytic network containing a cycle of length 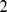 studied in [13]. One possible generalization without altering the original structure too much is the cycle of length 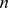 used here. We also included certain reverse reactions which often occur. On the one hand, we chose the simplest generalization (many more are thinkable), on the other hand this variant still matches closely to biological applications. The positive feedback provided by the cycle is crucial for the existence of a positive fixed point and its stability in all cycle variants we have discussed. We emphasize that in the particular autocatalytic network discussed, a positive and not a negative feedback leads to the stability property of the fixed point. This is due to the fact that the molecular species are all degrading. Therefore there needs to be a positive feedback to replace the vanished molecules. In our study a cycle provides the structure for the network to reproduce all the molecules and not only one type by a single positive feedback.

There are many biological applications that have similar cycles to those presented in this study, e.g. MAPK (mitogen-activated protein kinase) cascades and cell-cycle control models. We have used two examples of mitotic control mechanisms, namely the Spindle Assembly (SAC) and Position (SPOC) Checkpoints. Both checkpoints have high importance in cancer research [46], [56]. We took advantage of the well studied models on SAC [33]–[35] while for SPOC, we built a very simple model that is able to grasp the basic SPOC mechanism. Our analysis of these two models is consistent with our theory and shows that the feedback guarantees a positive stable fixed point. Additionally, the published models did neglect all productions and degradations reactions for all proteins and complexes. These reactions could be important for the effect on the model's prediction. For instance in a SAC model, neglecting these reactions led to an underestimate of the effect of the feedback loop at least with respect to the stability of the system [34].

As future work we aim at the inclusion of non-stoichiometric information, such as the kinetic rate relations or relative molecular concentrations. These might allow us to establish concrete results about the existence and stability of fixed points, and of other stationary regimes of higher dimension, such as periodic orbits and limit cycles [17]. Indeed, a novel molecular species (or a set of them) introduced to a reaction network can reinforce or breakdown the stability of a chemical system.

## Appendix

### Chemical Organization Theory

Let 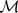 be a set and 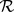 be a subset of 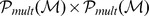 where 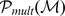 denotes the set of multisets over 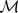. The pair 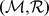 is called *reaction network* and we call 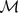 the set of *molecules* (or *species*) and 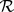 the set of *reactions*.

Like in chemistry, for 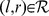 we also write 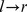 or
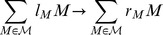
where we denote by 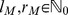 the multiplicity of 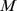 in 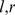 respectively. A reaction 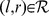 *occurs* when the molecules in 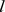 are *consumed* to *produce* the molecules in 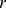 (what this actually means has to be made precise for every dynamics separately, e.g. the application of a reaction in stochastic dynamics is different from continuous dynamics).

Furthermore we define the *support* and the *product* of 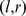 by
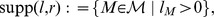


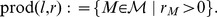



Let 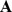 be a subset of 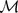. We define 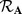, the set of reactions applicable to 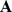, by setting
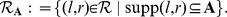



Abusing notation we use a reaction 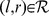 as an index as well and define the *stoichiometric matrix* 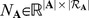 for 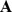 by
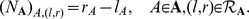




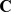
 being closed means that by applying reactions from 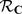 we do not get molecules outside 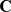. Formally speaking, a subset 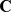 of 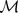 is *closed* if for all reactions 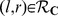 we have 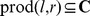, i.e. if 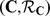 is a reaction network [14].


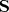
 being semi-self-maintaining means that if a reaction application consumes a species, there is a reaction producing this species. More formally, a subset 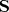 of 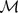 is *semi-self-maintaining* if for every 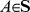 and 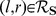 with 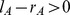 there is a 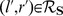 with 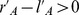.

A subset of 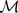 is a *semi-organization* if it is closed and semi-self-maintaining.

Semi-organizations entail a topological (non-stoichiometric) notion of stability in reaction networks. On the one hand, the closure property ensures that a semi-organization will not produce novel species. On the other hand the semi-self-maintenance ensures that the species that are consumed within the network are also produced. However, although semi-organization is a necessary condition for the occurrence of a fixed point, semi-organizations cannot be maintained over time in general, i.e. consider the reaction network 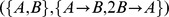 with the semi-organization 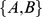.

In order to capture more of the notion of fixed states in a reaction network, we need to consider the stoichiometry of the reactions, and verify if the network can maintain itself, i.e. produce all the species at a higher or equal rate to what they are consumed.

A subset 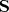 of 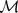 is *self-maintaining* if there is a vector 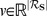 with strictly positive entries such that 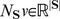 has only non-negative entries [16]. 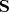 being self-maintaining means that all the reactions from 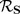 can occur at a certain strictly positive rate without decreasing the concentration of any species of 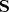.

A subset of 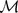 is a *chemical organization* if it is closed and self-maintaining [14], [16].

The term organization refers to a more stringent condition than semi-organization. Indeed, every organization is a semi-organization [16]. Moreover, organizations bridge the reaction network with its dynamical behavior. Given a reaction network and a kinetic law (e.g. mass action kinetics), it has been proven that the fixed points of the obtained ordinary differential equation (ODE) system, relate to the set of organizations as follows. For every fixed point the set of molecules with concentration higher than zero in that fixed point is an organization of the reaction network [16], [17].

### Fixed Point and Stability Analysis for the Particular Autocatalytic Network

The stoichiometric matrix 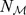 of the system 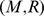 with the generalized cycle (case 1) defined in Section “A Particular Autocatalytic Network” is
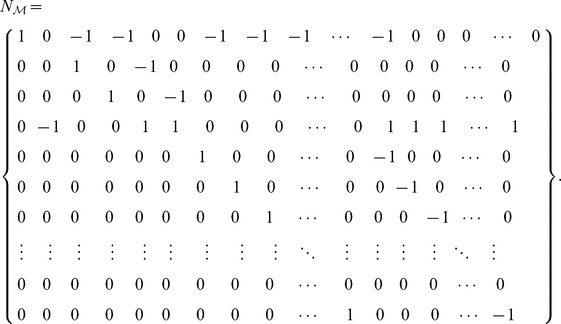
It is possible to guess a suitable vector to show the self-maintenance property of the whole system. For 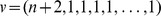 we get 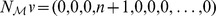. Hence the reaction network is an organization.

In a similar fashion we see that the full network is not an organization in the cases 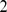, 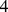, 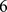 since the row 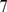 is missing and species 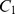 can no longer be maintained. In case of the cycle with reversible reactions (cases 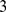, 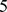) we also find the network to be an organization with the help of a similar argument. More precisely, the stoichiometric matrix needs to be enlarged by the reverse reactions and the flux vector slightly adjusted (see Figure 4 for typical Hasse diagrams of these cases).

For the determination of fixed points and their stability we employ the ODEs given by mass action kinetics for the reaction networks. The reaction rate constants we did not explicitly define are assumed to be all equal to 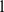.

The ODEs can be written down for all six cases at once using the parameter 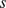 which is equal to 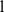 if the cycle is closed (cases 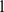, 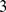, 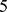) and equal to 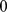 if open (cases 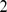, 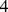, 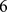). We also use the constant 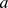 for the rate of the reverse reactions.

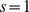
, 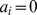 – generalized cycle

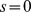
, 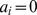 – generalized open cycle (the feedback is missing)

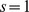
, 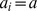 – generalized cycle with reversible reactions

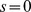
, 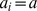 – generalized open cycle with reversible reactions (the feedback is missing)

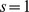
, 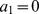, 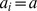 – generalized cycle with all reactions reversible except of one

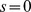
, 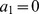, 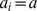 – generalized open cycle with all reactions reversible (the feedback is missing) except of one

The last section already showed that there is no fixed point in the cases 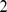, 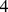, 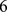. Only case 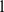 can be treated analytically. For the cases 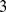, 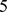 numerical simulations can be done.

The ODEs for our reaction network are given by
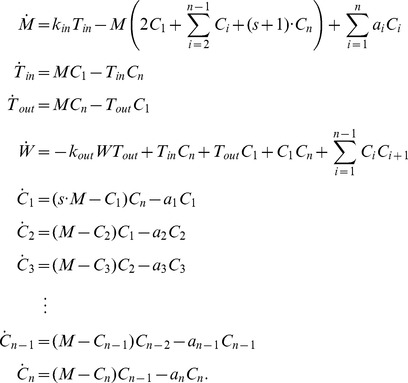



Its Jacobian matrix is
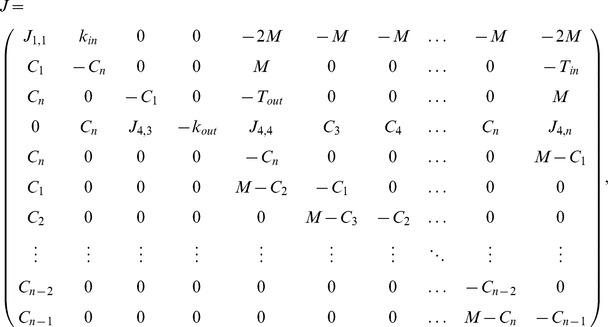
where 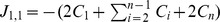, 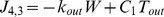, 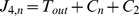, and 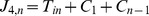.

For case 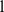 we look for positive steady states and set the system to 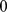. We immediately get the following relations:
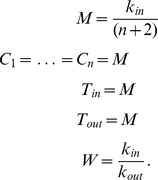
To determine the stability we use the Jacobian matrix which is in the fixed point given by
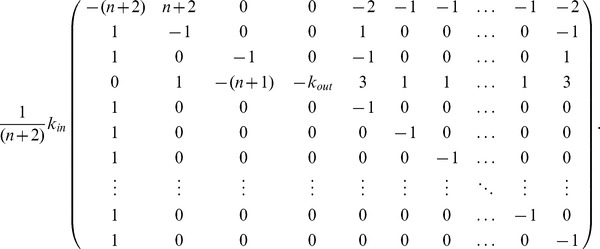
After tedious computations we can find that the eigenvalues of the Jacobian are as follows
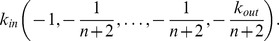
This means that the fixed point we found is stable. For the remaining cases 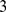, 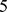 we employ a parameter scan and numerically (with Mathematica [26]) compute fixed points and their stability. For the case 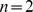 we used the following pairs of parameter values
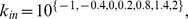


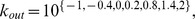


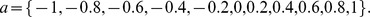
We could verify that in these cases the results were as expected. Apparently the effects of the parameters are neglectable. All numerical simulations of cycle variants as ODEs (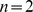) show the same expected results (Figure 5).
